# Decrease in survival and fecundity of *Glossina palpalis gambiensis* vanderplank 1949 (*Diptera*: *Glossinidae*) fed on cattle treated with single doses of ivermectin

**DOI:** 10.1186/1756-3305-6-165

**Published:** 2013-06-06

**Authors:** Sié H Pooda, Karine Mouline, Thierry De Meeûs, Zakaria Bengaly, Philippe Solano

**Affiliations:** 1 Ministère des Ressources Animales et Halieutiques, PATTEC-PCZLD, Ouagadougou, Burkina Faso; 2 Institut de Recherche pour le Développement (IRD), UMR 224 MIVEGEC, Ouagadougou, Burkina Faso; 3 Institut de Recherche en Science de la Santé (IRSS), Ouagadougou, Burkina Faso; 4 Centre International de Recherche Développement sur l’Elevage en zone Subhumide (CIRDES), Institut de Recherche pour le Développement (IRD), UMR 177 IRD-CIRAD INTERTRYP, 01 BP 454, Bobo-Dioulasso, Burkina Faso; 5 Centre International de Recherche Développement sur l’Elevage en zone Subhumide (CIRDES), 01 BP 454, Bobo-Dioulasso, Burkina Faso

**Keywords:** Ivermectin, *Glossina palpalis gambiensis*, Survival, Fecundity, Cattle, Burkina Faso

## Abstract

**Background:**

Human and Animal Trypanosomes are major problems for the socio-economic growth of developing countries like Burkina Faso. Ivermectin is currently used to treat humans in mass drug administration programs in Africa, and is also commonly used for veterinary purposes. In this study, we tested the effect of ivermectin injected into cattle on the survival and fecundity of *Glossina palpalis gambiensis*, the main vector of human and animal trypanosomes in West Africa.

**Methods:**

Three cows (local zebu*baoulé crossbreds) were used, and received either no ivermectin (for the control), or ivermectin at therapeutic dose (0.2 mg/kg) and 10 times the therapeutic dose (2 mg/kg) respectively. *G*. *palpalis gambiensis* were fed on the cattle for their first bloodmeal, and then either on cattle or on membrane for subsequent meals.

**Results:**

Our results showed that survival of *Glossina palpalis gambiensis* was significantly decreased when they were fed on cattle treated with ivermectin. This decrease in survival ranged from 21% to 83.7% for the therapeutic dose (0.2 mg/kg), up to 8 days after treatment. The effects of a dose of 2 mg/kg were higher with a 78.3% to 93.9% decrease in survival, until 14 days after injection. The therapeutic dose of ivermectin also decreased fecundity, and delayed the first larviposition, but there was no significant effect on hatching rate.

**Conclusion:**

Ivermectin injected into cattle may constitute an additional potential tool for the control of *Glossina palpalis gambiensis* and possibly other vector species. Further studies will be needed to assess its effect on trypanosome transmission, and to define more precisely the adequate dose to be used for control purposes.

## Background

Tsetse flies (*Diptera*: *Glossinidae*) are the main vectors of trypanosomes (*Kinetoplastida*: *Trypanosomatidae*), which cause human and animal trypanosomiasis in Sub-Saharan Africa (HAT and AAT respectively). For HAT, vector control is an important complement to case detection and treatment, because reducing vector density can rapidly halt human trypanosomiasis transmission [1,2]. Also, in the absence of any chemoprophylaxis, vector control remains the only available strategy capable of protecting human individuals from acquiring infection [3]. For AAT, vector control remains widely used. Tsetse populations may be reduced using a variety of techniques, including insecticide impregnated traps and targets, live-baits, sequential aerial spraying, and sterile male releases [4,5]. Despite the existence of such tools, human and animal trypanosomes are still having an enormous impact on public health and economic development of Sub-Saharan Africa.

With the objective of finding additional simple tools for vector control, ivermectin, a drug widely used in human and veterinary medicine, has been preliminarily reported to have some effect on the life history traits of several vector species, including anopheles mosquitoes and tsetse flies [6,7]. However, so far, most of the experiments conducted on tsetse have used *in vitro* blood-feeding assays, or have considered non-natural hosts (guinea pigs, rabbits, etc.). Hence, the question on whether these results can be extrapolated to the field using the natural host-vector system still remains. In the present study, we investigated for the first time the effects of ivermectin on epidemiologically relevant life history traits (survival and fecundity) of *Glossina palpalis gambiensis*, which is the main vector of HAT and AAT in West Africa [8]. In addition, to be as close as possible from realistic field conditions, two doses of ivermectin, including the recommended therapeutic dose, were injected to local cattle breeds on which the tsetse flies were fed.

## Methods

This study was conducted at the “Centre International de Recherche Développement sur l’Elevage en zone Subhumide” (CIRDES), in Bobo-Dioulasso, Burkina Faso, which has a mass rearing facility of three species of tsetse, including *G*. *palpalis gambiensis* (approximately 140 000 female flies and 5 000 pupae per day).

### Materials

Teneral males and females *Glossina palpalis gambiensis* (3 days old) and mature males (6 days old, for mating) were used. They were maintained in Roubaud cages [9], at a maximum density of 35 flies per cage. The climatic conditions were 25 ± 1°C temperature and 70 ± 5% relative humidity. For mating, mature tsetse males were introduced in female cages at a ratio of 1 male for 3 females. Three crossbred cows (Zebu X Baoulé taurine), 3 years old and 110 kg mean weight, originating from surrounding farms, were used. On arrival, each cow was treated using diminazene aceturate (3.5 mg/kg) and albendazole, and the experiments began 15 days after this treatment.

Ethical approval of this experiment has been given from the CIRDES ethic committee.

### Experimental design

The experiments started by single subcutaneous injection of ivermectin (IVOMEC D®, Merial) [10] into two cows. The recommended therapeutic dose (0.2 mg/kg) and a high dose of 2 mg/kg (10 times the therapeutic dose) were tested. The high dose of 2 mg/kg was used in order to ensure that a minimum effect of ivermectin on *G*. *palpalis gambiensis* was observed, due to the absence of preliminary data. The third cow was used as control and therefore did not receive any dose of ivermectin.

Tsetse flies were fed every 48 h by applying the cages to both sides of the cattle. The cages were maintained using rubber, covered with black fabric in order to create darkness around the tsetse, allowing them to take their bloodmeal. Only engorged flies were monitored, *i*.*e*. a total of 841 tsetse. These numbers were allotted as can be seen in Table 1. Tsetse that were given a single blood meal from cattle were fed thereafter *in vitro* via membrane feeding according to the classical method of feeding in the insectarium [11].

**Table 1:** Number of tsetse monitored according to ivermectin dose, day post injection, and number of bloodmeals on cow

**Dose of IVOMEC**	**Control**				**0,2 mg/kg**				**2 mg/kg**				**Total**
**Blood meal on cow**	**Single**		**Several**		**Single**		**Several**		**Single**		**Several**		
**Sex**	**M**	**F**	**M**	**F**	**M**	**F**	**M**	**F**	**M**	**F**	**M**	**F**	
Number D2	25	23	24	20	25	21	25	20	21	20	25	15	264
Number D8	22	24	25	25	24	25	23	25	24	24	25	25	291
Number D14	25	20	25	21	25	24	24	24	24	25	25	24	286
**Total**	**72**	**67**	**74**	**66**	**74**	**70**	**72**	**69**	**69**	**69**	**75**	**64**	**841**

D2, D8 and D14: 2, 8 and 14 days after injection of ivermectin to cattle (start of experiment).

*M*, males; *F*, females.

“single” and “several” refer to the number of bloodmeals on cow.

### Data analysis

Statistical analyses were performed using the software R 2.13.1 [12]. Based on daily mortality, we performed survival curves of Kaplan and Meier for the different treatments. The Wilcoxon test was used to compare survival curves between the different treatments. The level of significance was p<0.05. The median survival (MS) was used to determine the percentage of lifetime reduction as follows:

$$\begin{array}{ll} \;\%\mathrm{reduction}= & 100*\left(\mathrm{MS}\;\text{of}\;\text{control-MS}\;\text{of}\;\text{treatment}\right)\\ & /\mathrm{MS}\;\text{of}\;\text{control} \end{array}$$

Fecundity (average number of larvae produced in every cage divided by number of females per cage) and hatching rates (percentage of emerging adults from the pupae for every cage) were compared between treatments, for the females surviving until maturity. These parameters were calculated from the 20th day after the first blood meal (corresponding to the mean age of the first larviposition) and the following larvipositions (every 10 days). A one way ANOVA in which all data were weighted by the initial number of females in the corresponding cage was used to compare fecundity between different treatments. Hatching rate between treatments was compared using the chi-square test of independence. The sequential Bonferronni procedure was applied for multiple testing [13].

## Results

### Effects of different doses of ivermectin on tsetse survival

#### Survival of tsetse fed on the control cow

**Figure 1:**
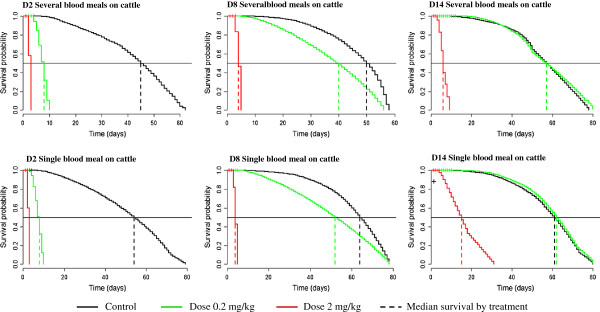
**Survival curve of tsetse in experiment according to ivermectin dose, number of bloodmeals on treated cattle, and time after ivermectin injection (2 days = D2, 8 days = D8 and 14 days = D14).**

Survival curves between treatments compared to the survival of the control are shown in Figure 1. Survival curves are presented according to the number of bloodmeals taken on cattle (one versus several). For the control, median survival time of tsetse which were given several blood meals was 45, 50 and 57 days, respectively for days 2, 8 and 14 after ivermectin injection, hence an average of 50 days. For tsetse which were given one single blood meal on the control cattle, median survival time was 54, 64 and 61 days for the same periods, hence an average of 60 days.

#### Survival of tsetse fed on the cow treated with a dose 0.2 mg/kg of ivermectin

For the tsetse having fed several times on injected cattle, the median survival time was 8, 40 and 57 for 2, 8 and 14 days post feeding on the ivermectin treated cow respectively (see Figure 1). The effect of 0.2 mg/kg of ivermectin was similar whether the tsetse took all their blood meals or just one on the treated cow (i.e. 8, 52 and 62 days respectively).

The survival comparisons between the tsetse fed on cattle treated with this dose and the tsetse fed on the control, gave a significant reduction in tsetse longevity, from 83.7% at day 2 post ivermectin treatment, to 21% until 8 days after ivermectin injection (*P*=0 for both). There was no longer an effect at day 14 post injection (Figure 1).

#### Survival of tsetse fed on the cow treated with a dose of 2 mg/kg of ivermectin

The survival curves of tsetse exposed to this dose (2mg/kg) are presented in Figure 1. Tsetse that had taken several blood meals on the cattle treated at this dose had a median survival time of 3, 4 and 6 days respectively at 2, 8 and 14 days after treatment. For tsetse that had taken a single blood meal on cattle treated at this dose, survival time was 3, 4 and 15 days respectively. Finally, regardless of the number of blood meals taken on the treated cattle, the 2 mg/kg dose induced mortality of all tsetse at 3, 5 and 31 days after the first blood meal, respectively at 2, 8 and 14 days after ivermectin treatment. This gives a significant survival reduction of 93.9%, 92.9% and 78.3% respectively for 2, 8 and 14 days after treatment (*P* =0).

### Effect of different doses of ivermectin on tsetse fecundity

The effect of ivermectin was measured only with the tsetse from 8 and 14 days after injection, and only with tsetse fed on the cattle used as control and the cattle injected with the therapeutic dose, since the other tsetse were killed by ivermectin before maturity, without having laid any larva.

The control cow had a fecundity of 3.59 pupae per female at D8 and for the 80 days follow-up (See Table 2), in comparison to 2.35 pupae per female for tsetse exposed to the therapeutic dose at D8. This corresponds to a significant decrease of 34.5% (*P* = 0.04 with sequential Bonferronni correction). Total fecundity of tsetse fed on the control cow at D14 (3.77 pupae/female) was also higher in comparison to those from the treated cows (2.89 pupae/female), but the 23% decrease in fecundity was not significant (*P*=0.80). Our results also showed a delay for the day of first larviposition due to the effect of the therapeutic dose at 8 days post injection, which rose from 15 to 19 days (see Table 2).

**Table 2:** **Day of first larviposition, fecundity, and hatching rate of *Glossina palpalis gambiensis *according to ivermectin dose, and day post injection**

**Time after ivermectin injection**	**Treatment**	**Day of 1st larviposition**	**Fecundity at day:**							**Hatching rate (%)**
			**20**	**30**	**40**	**50**	**60**	**70**	**80**	
D8	Control	15	0.71 ^a^	1.51 ^a^	2.57^a^	3.12^a^	3.39^a^	3.53^a^	3.59^a^	73.30^a^
	Dose 0.2 mg/kg	19	0.10 ^a^	0.70 ^a^	1.45^b^	1.70^a^	2.10^b^	2.20^b^	2.35^b^	78.72^a^
D14	Control	16	0.49^a^	1.85^a^	2.85^a^	3.23^a^	3.41^a^	3.59^a^	3.77^a^	82.99^a^
	Dose 0,2 mg/kg	15	0.37 ^a^	1.46^a^	2.15^a^	2.41^a^	2.65^a^	2.80^a^	2.89^a^	78.95^a^

Fecundity is the average number of larvae produced in every cage divided by number of females per cage. Hatching rate is the percentage of emerging adults from the pupae for every cage (see Methods for details). In the same column, values with different letters are significantly different. For the different days, only D8 and D14 were considered because all the tsetse of D2 were killed by ivermectin treatment.

We did not observe significant differences between hatching rates of the tsetse fed on the control cow versus the ones fed on ivermectin injected cows (see Table 2).

## Discussion

### Effects of ivermectin on tsetse survival and longevity

These results show for the first time that the therapeutic dose of ivermectin injected into cattle reduces the survival and lifespan of *G*. *palpalis gambiensis*. The effect can be observed up to 8 days after injection of cattle, but seems to disappear after 14 days.

The 10 times therapeutic dose (2 mg/kg), showed the highest toxicity with a mean reduction of 86% in survival compared to controls, and lasted up to 14 days after injection. Distelmans *et al*. [6] had observed a lethal effect of ivermectin up to the dose of 2 mg/kg on *G*. *palpalis palpalis*, but with tsetse fed on guinea pigs and goats neither a 0.5 mg/kg nor 1 mg/kg dose had any effect on the survival of *G*. *palpalis palpalis* fed on guinea pigs [14], similarly no effect was seen in *G*. *tachinoides* fed on pigs [15]. These differences, as compared to our results, could be due to variation in the kinetics of the molecule in the different host species (guinea pig, goat, pig) studied by these authors, or to a change in the current formulation of the drug. In our study, a single blood meal on an animal treated with ivermectin was enough to induce mortality on tsetse fed on cattle, up to 8 days after treatment. Distelmans *et al*. [6] obtained an effect of longer duration (16 days after injection) but using higher doses of ivermectin (10 mg/kg).

### Effects of ivermectin on the fecundity of *Glossina palpalis gambiensis*

Our results show that the therapeutic dose of ivermectin resulted in a 20-30% reduction of the fecundity of surviving flies. Contrary to our observations, Van Den Abbeele *et al*. [16] found that the therapeutic dose of ivermectin did not affect the fertility of *G*. *palpalis palpalis* fed on rabbits. Nevertheless, they found that ivermectin at a dose of 0.5 mg/kg caused a decline in *G*. *p*. *palpalis* fertility (number of pupae and pupae weight average) [14]. We also showed that the date of the first larviposition was delayed. In line with other studies [14], we observed no effect of ivermectin on the hatching rate at D8 and D14. A decrease in the fecundity of *G*. *morsitans* fed on a cow treated with ivermectin at a dose of 0.2 mg/kg had also been reported by Langley and Roe [17]. They reported a 44% decrease observed 7 days after drug administration (until the second ovarian cycle), followed by a gradual return to normal fecundity. The effect of ivermectin on the fecundity of flies can be explained by different factors: a delay in the ovulation process, an increase in the duration of gestation, and/or a disruption of pupation [16].

### Effect of ivermectin on trypanosome transmission

Basically, our results on *G*. *palpalis gambiensis* are in agreement with those obtained using other models cited in the literature [6-15]. Thus, the decline in longevity and fertility will have an impact on tsetse populations (in particular density and age structure), which in turn may have consequences on the epidemiology of trypanosomiasis in treated zones. According to Pollock [8], the duration of development of trypanosomes in tsetse is on average 10 days for *T*. *vivax*, 12 to 14 days for *T*. *congolense* and 20 to 30 days for *T*. *brucei*. The reduced survival of tsetse using ivermectin should, therefore, impact negatively the development of all types of trypanosomes, especially *T*. *brucei*, including *T*. *b*. *gambiense*, which causes sleeping sickness (HAT) in West and Central Africa, and *T*. *b*. *brucei* also responsible of AAT. It has to be noticed that the effects of ivermectin mass treatments on humans have also been reported on other vector borne diseases: in Senegal, Kobylinski *et al*. [18] reported a decrease of *Plasmodium* infection rates in *Anopheles gambiae* in an area subjected to mass distribution of ivermectin.

### Potential use of ivermectin for tsetse control

This study shows that ivermectin, given its effects on tsetse longevity and fecundity, may constitute an additional tool for tsetse control. However, the limits of its use have also to be acknowledged. The cost of an ivermectin treatment at a therapeutic dose is 2100 cfa francs (~4.3 $) per treated animal (as a mean for a 250 kg animal) and may restrict its use if all cattle in an area were to be treated, but this would not necessarily have to be the case. In addition, the use of generics may decrease this cost. We show that the impact of ivermectin would be greater with a dose of 2 mg/kg. However, this dose is not recommendable because of the associated financial costs, and also because of side effects on cattle. Moreover, ivermectin has effects on non-target fauna [19,20], there are residues in milk when dairy cows are treated, and there is a risk of development of resistance to the molecule due to its mass use for human health [21].

However, interesting possibilities remain. Ivermectin is already being mass distributed to human populations to treat lymphatic filariasis and onchocerciasis [22]. In addition, it is also used by local farmers, with the intention to control internal parasites of their cattle such as nematodes [10]. Ivermectin could thus form part of an integrated development package. In areas where measures already exist to control trypanosomiasis (such as the PATTEC project in Burkina Faso which already uses impregnated traps and targets, and trypanocides on cattle), the idea would be to add the use of ivermectin to contribute to improving animal production.

## Conclusions

The results of this study show that ivermectin injected into cattle affects survival, lifespan, and fecundity of *Glossina palpalis gambiensi*s. The effect of the therapeutic dose on tsetse is maintained until 8 days after ivermectin injection. These results suggest that the mass use of ivermectin in the field for both human and animal health could have a significant impact on the transmission of vector-borne diseases such as trypanosomiasis, but also malaria or others. Thus avermectins in general, and in particular ivermectin, may constitute an additional tool for the control of trypanosomiasis in areas where the vectors mainly feed on domestic animals such as cattle. Further studies should focus on the direct effects of this molecule on the development of trypanosomes in tsetse, and should aim at determining the optimal dose to be used for control.

## Competing interests

The authors declare that they have no competing interests.

## Authors’ contributions

Designed the study: SHP, KM, TDM, ZB, PS. Made the experiments: SHP, KM. Analysed the results: SHP, TDM, PS. Drafted and corrected the ms: SHP, KM, TDM, ZB, PS. All authors read and approved the final version of the manuscript.
